# Retrospective Analysis of Hematological Parameter Changes in DMARD-Naive Rheumatoid Arthritis Patients Treated with Methotrexate: Correlation with Disease Activity and Treatment Outcomes

**DOI:** 10.3390/biomedicines14030625

**Published:** 2026-03-11

**Authors:** Esra Dilsat Imrak, İlknur Aktas

**Affiliations:** 1 Department of Rheumatology, Balıkesir Ataturk City Hospital, Balıkesir 10100, Turkey; 2 Department of Physical Medicine and Rehabilitation, University of Health Sciences, Fatih Sultan Mehmet Training and Research Hospital, Istanbul 34752, Turkey

**Keywords:** rheumatoid arthritis, methotrexate, hematological parameters, disease activity, treatment response, biomarker

## Abstract

**Background/Aim:** This study aimed to evaluate the changes in hematological indices following methotrexate (MTX) initiation and assess their correlation with and predictive value for treatment responses in rheumatoid arthritis (RA) patients. **Methods:** A retrospective study was conducted on 299 DMARD-naïve RA patients who received MTX monotherapy for 12 weeks. Univariate and multivariate logistic regression identified predictors of remission and low disease activity. Correlation analyses assessed relationships between hematological and disease activity changes. Receiver operating characteristic (ROC) curve analysis evaluated the discriminatory ability of hematological parameters. **Results:** After 12 weeks of MTX, significant decreases were observed in white blood cell (*p* = 0.025), neutrophil (*p* = 0.026), hemoglobin (*p* = 0.001), and platelet counts (*p* < 0.001), alongside an increase in red cell distribution width (RDW) (*p* < 0.001). Multivariate analysis identified only baseline DAS28-CRP (OR: 9826.7, *p* < 0.001) and CRP (OR: 0.45, *p* = 0.005) as independent predictors for remission, and baseline swollen joint count, DAS28-CRP, and CRP for LDA. Hematological parameters were not independent predictors. ROC analysis revealed neither baseline values nor changes in hematological indices had satisfactory discriminatory power for remission or LDA. **Conclusions:** Hematological parameter changes do not serve as robust independent predictors for early treatment response. Clinical disease activity indices remain superior for prognostication in DMARD-naïve patients starting MTX.

## 1. Introduction

Rheumatoid arthritis (RA) is the most common form of chronic inflammatory polyarthritis, with increasing incidence worldwide [1]. It is characterized by progressive synovial inflammation, leading to joint destruction, anatomical deformity, and significant disability [2]. The primary therapeutic goal in RA is to suppress inflammatory disease activity, aiming for clinical remission or at least low disease activity to prevent structural damage and preserve function [3].

The pathogenesis of RA involves a complex network of immune dysregulation and cytokine overproduction. Proinflammatory cytokines such as tumor necrosis factor-alpha (TNF-α), interleukin-6 (IL-6), IL-17, monocyte chemoattractant protein-1 (MCP-1), and interferons are markedly elevated in patients with RA and drive both local joint inflammation and systemic manifestations [4]. These cytokines not only activate synovial fibroblasts and promote angiogenesis but also exert profound effects on hematopoiesis and the function of circulating blood cells [5]. For instance, IL-6 is a key mediator of the acute-phase response, anemia of chronic disease, and thrombocytosis [6]. Immune cells, including lymphocytes, monocytes, and neutrophils, are central to this inflammatory cascade, and their circulating numbers and ratios are increasingly recognized as dynamic reflections of systemic immune activity [7]. Furthermore, emerging evidence indicates that even erythrocytes and platelets participate in cytokine signaling and storage, underscoring the integral role of the entire hematological system in the inflammatory state of RA [8,9].

In clinical practice, disease activity in RA is assessed using a combination of clinical evaluation and laboratory biomarkers. Conventional biomarkers such as rheumatoid factor (RF), anti-cyclic citrullinated peptide antibody (ACPA), C-reactive protein (CRP), and erythrocyte sedimentation rate (ESR) are widely utilized [10]. However, their diagnostic and monitoring utility has notable limitations. Sensitivities for RF, ACPA, CRP, and ESR vary considerably, while their specificities are suboptimal due to elevations in other inflammatory, infectious, or autoimmune conditions [11]. Moreover, clinical scoring systems like the 28-joint Disease Activity Score (DAS28) incorporate subjective components and may not fully capture the underlying inflammatory burden, particularly in cases where acute-phase reactants remain discordantly normal despite active synovitis [12]. This discrepancy highlights the need for more reliable, accessible, and objective biomarkers to complement existing tools.

Consequently, there has been growing interest in hematological indices derived from routine complete blood counts (CBC) as potential biomarkers of inflammation and disease activity in RA. Parameters such as the neutrophil-to-lymphocyte ratio (NLR), platelet-to-lymphocyte ratio (PLR), mean platelet volume (MPV), and hemoglobin (Hb) levels have been associated with disease activity in cross-sectional studies [13,14]. These indices are attractive due to their low cost, wide availability, and objective nature. However, existing evidence is predominantly cross-sectional, often lacks pretreatment baseline data, and does not adequately account for the confounding effects of specific therapies.

Methotrexate (MTX) is the established first-line anchor disease-modifying antirheumatic drug (DMARD) in the treatment of RA [3]. Its mechanism, while incompletely understood, includes anti-inflammatory and immunomodulatory effects mediated partly through folate pathway inhibition. Notably, MTX can directly influence hematopoiesis, potentially altering red blood cell indices, hemoglobin levels, and possibly other cellular lineages, even with folate supplementation [15]. Therefore, longitudinal assessment of hematological parameters in DMARD-naïve patients initiating MTX is essential to distinguish treatment-induced hematological changes from those reflecting modulation of disease activity.

To date, no study has comprehensively evaluated the longitudinal changes in a broad panel of hematological parameters—including cellular counts, ratios, and erythrocyte indices—in DMARD-naïve RA patients starting MTX, nor has one examined their correlation with dynamic changes in disease activity and their potential to predict early treatment outcomes such as remission or low disease activity.

Therefore, this retrospective cohort study aimed to characterize the changes in hematological parameters after 12 weeks of MTX monotherapy in DMARD-naïve RA patients, investigate the correlation between these hematological changes and changes in conventional disease activity measures (DAS28-CRP, CRP), and assess whether baseline or on-treatment hematological parameters can serve as predictive biomarkers for achieving remission or low disease activity at 12 weeks. By addressing these objectives, this study seeks to clarify the hematological footprint of early MTX therapy and explore the utility of routine blood parameters as complementary tools for monitoring treatment response in RA.

## 2. Materials and Method

### 2.1. Study Design and Participants

This single-center retrospective study included 299 patients diagnosed with RA between June 2024 and June 2025 at Balıkesir Ataturk City Hospital, a tertiary care center. The study was reviewed and approved by Instutional Review Board for Ethics in Human Research (protocol number: 2025/11/128).

DMARD-naive RA patients aged > 18 years who fulfilled the American College of Rheumatology/European Alliance of Associations for Rheumatology (ACR/EULAR) 2010 classification criteria for RA [16] were recruited between June 2024 and June 2025.

The inclusion criteria were: (1) DMARD-naïve status at baseline (no prior conventional synthetic, biologic, or targeted synthetic DMARD use), (2) initiation of methotrexate (MTX) as the first-line monotherapy, (3) availability of complete clinical and laboratory data at baseline (Week 0) and at the 12-week follow-up visit. Exclusion criteria included: (1) concomitant use of other DMARDs or corticosteroids at a dose > 10 mg/day prednisone equivalent within 3 months of baseline, (2) presence of other inflammatory diseases (e.g., systemic lupus erythematosus, active infection), hematological disorders, malignancy, GFR (glomerular filtration rate) < 30 mL/min/1.73 m^2^, (3) pregnancy or lactation.

### 2.2. Data Collection

Demographic and clinical data were extracted from patient records. The number of tender and swollen joints, the visual analog scale (VAS) scores, and the physician’s global assessment of disease were recorded. Swollen joint count (SJC) and tender joint count (TJC) each constitute 28 joint counts. Visual analog scale (VAS) and Patient Global Assessment of Disease Activity (PGA) were assessed on a 10 cm visual analog scale.

Rheumatoid factor (RF) levels were measured using nephelometric analysis (Siemens Dade Behring N2, Marburg, Germany), with values above 20 IU/mL considered positive according to the manufacturer’s instructions. Anti-cyclic citrullinated peptide (anti-CCP) antibodies were measured by enzyme-linked immunosorbent assay (ELISA) (Cobas e411, Roche Diagnostics, Mannheim, Germany), with levels above 5 IU/mL defined as positive. Complete blood count analysis were performed using an automated hematology analyzer (Sysmex XN 1000, Kobe, Japan). Serum C-reactive protein (CRP) concentrations were determined using ELISA kit (Cobas e411 and Cobas e601, Roche Diagnostics, Mannheim, Germany). CRP concentrations were expressed in mg/dL. Seropositive RA was used for patients who have positive results for either RF or anti-CCP.

Hematological parameters were obtained from complete blood count (CBC) analyses performed using standard automated hematology analyzers. The following parameters were recorded: white blood cell count (WBC), absolute neutrophil count (NEU), absolute lymphocyte count (LYM), absolute monocyte count (MONO), hemoglobin concentration (HGB), platelet count (PLT), mean platelet volume (MPV), and red cell distribution width (RDW). Derived inflammatory ratios were calculated: neutrophil-to-lymphocyte ratio (NLR = NEU/LYM) and platelet-to-lymphocyte ratio (PLR = PLT/LYM). All clinical and laboratory assessments were repeated at the 12-week follow-up visit.

Disease activity was measured by DAS28-CRP [17] scores. Remission and low disease activity (LDA) were assessed using American College of Rheumatology criteria in both groups after 24 weeks [18]. Disease activity was categorized by remission as DAS28-CRP < 2.6 and low disease activity as DAS28-CRP 2.6–3.2 at the 24th week of treatment.

### 2.3. Statistical Analysis

Data were analyzed using IBM SPSS Statistics for Windows, version 20.0 (IBM Corp., Armonk, NY, USA). Continuous variables were tested for normality using the Kolmogorov–Smirnov test. Non-normally distributed data were presented as median and interquartile range (IQR), and normally distributed data as mean ± standard deviation (SD). Categorical variables were presented as frequencies and percentages. Comparisons between two subgroups were performed using the nonparametric Mann–Whitney U test.

Baseline characteristics were summarized descriptively. Changes in hematological and clinical parameters from baseline to week 12 were compared using the Wilcoxon signed-rank test for non-normally distributed paired data. Univariate logistic regression analysis was performed to identify factors (demographic, clinical, and baseline hematological parameters) associated with the achievement of remission and LDA. Multiple linear regression analysis was used to assess whether changes in hematological parameters could predict the outcomes (remission/LDA). Multicollinearity among variables in the multivariate models was assessed using the Variance Inflation Factor (VIF), with a VIF > 5 indicating potential issues.

Correlations between laboratory variables were evaluated using Spearman’s or Pearson’s correlation coefficients. Linear regression analysis was performed to identify independent hematological predictors of DAS28-CRP improvement, with ΔDAS28-CRP as the dependent variable and changes in all hematological parameters (ΔWBC, ΔNEU, ΔLYM, ΔNLR, ΔMONO, ΔHGB, ΔPLT, ΔPLR, ΔMPV, ΔRDW) as independent variables. Receiver operating characteristic (ROC) curves were constructed for significant hematological parameter changes, and the area under the curve (AUC) was calculated to determine the optimal cutoff value for each parameter. A *p*-value < 0.05 was considered statistically significant.

A post hoc power analysis was conducted based on the observed effect sizes. With a sample size of 299, the study achieved >80% power to detect correlations with ≥r 0.18 at α = 0.05. However, power was limited (<80%) for detecting small effect sizes (Cohen’s d < 0.2) in paired comparisons.

## 3. Results

A total of 299 DMARD-naïve RA patients were included in this retrospective analysis. The baseline characteristics of the study population are presented in Table 1. The cohort was predominantly female (72.6%) with a median age of 57 years (IQR 47–65). The median symptom duration was 0.5 years, and 70.7% of patients were seropositive. Mean DAS28-CRP change was 0.59 ± 0.33 after 12 weeks of treatment. Remission was achieved in 55 patients (18.3%), and low disease activity was achieved in 168 patients (56%).

### 3.1. Hematological Parameter Changes Following MTX Treatment

Comparative analysis of hematological parameters at baseline and week 12 revealed significant alterations (Table 2). A statistically significant decrease was observed in WBC (*p* = 0.025), NEU count (*p* = 0.026), HGB level (*p* = 0.001), PLT count (*p* < 0.001), PLR (*p* = 0.011), and a significant increase in RDW (*p* < 0.001). In contrast, no significant changes were found in LYM count, NLR, MONO count, or MPV.

Subgroup analyses were performed to assess the homogeneity of the study population. Comparisons based on gender and seropositivity status revealed no statistically significant differences in baseline hematological parameters, disease activity scores (DAS28-CRP, CRP), or treatment outcomes (change in activity scores, remission, LDA) between groups (all *p* > 0.05, Table 3).

Stratified analyses were performed to assess the impact of seropositivity. Baseline demographic, clinical, and hematological parameters were largely similar between seropositive (*n* = 217) and seronegative (*n* = 82) patients, although symptom duration was slightly longer in the seropositive group (*p* = 0.003, Supplementary Table S1). Furthermore, the changes in hematological parameters and disease activity scores from baseline to week 12 did not differ significantly between the two groups (Supplementary Table S2). During the study period, no methotrexate-related myelosuppression was observed.

During the study period, no methotrexate-related myelosuppression was observed in the patients included in the study.

### 3.2. Factors Associated with Treatment Response

Univariate and multivariate logistic regression analyses were performed to identify factors associated with achieving remission and LDA at week 12 (Table 4). In univariate analysis, baseline TJC, SJC, DAS28-CRP, and CRP were significantly associated with both outcomes. In the subsequent multivariate model, which was checked for multicollinearity (all VIF < 2, Supplementary Table S3), only baseline DAS28-CRP (OR: 9826.703, 95% CI: 179.78–537,099.4, *p* < 0.001) and CRP (OR: 0.452, 95% CI: 0.261–0.782, *p* = 0.005) remained independent predictors for remission. For LDA, baseline SJC (OR: 1.803, 95% CI: 1.34–2.42, *p* < 0.001), DAS28-CRP (OR: 0.168, 95% CI: 0.052–0.538, *p* = 0.003), and CRP (OR: 1.47, 95% CI: 1.21–1.79, *p* < 0.001) were independent predictors. None of the hematological parameter changes showed significant independent association with either outcome in the multivariate analysis.

### 3.3. Correlation Between Hematological Changes and Disease Activity Improvement

**Figure 1:**
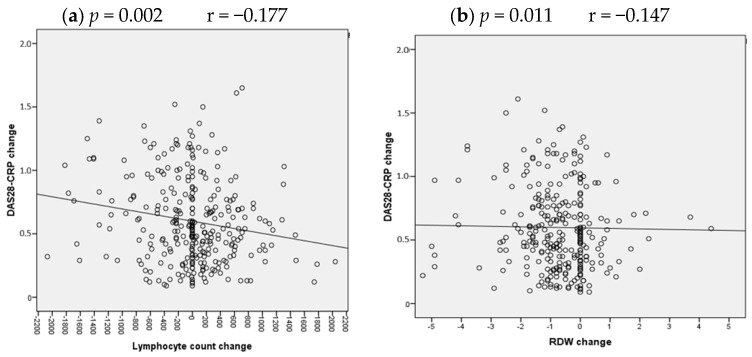
Spearman’s correlation analysis was applied to estimate relationships between DAS28-CRP changes and hematological parameters in RA patients. Scatter plot correlations of DAS28-CRP change with lymphocyte count change (**a**) and RDW change (**b**) after 12 week methotrexate treatment. The linear regression equation for (**a**) is: ΔDAS28-CRP = 0.589 − 0.00024(ΔLymphocyte), R^2^ = 0.031, *p* = 0.001. For (**b**): ΔDAS28-CRP = 0.589 − 0.004(ΔRDW), R^2^ = 0.022, *p* = 0.006. Correlation coefficients and *p*-values are presented in every scatter plot. *p* values < 0.05 were considered statistically significant.

**Figure 2:**
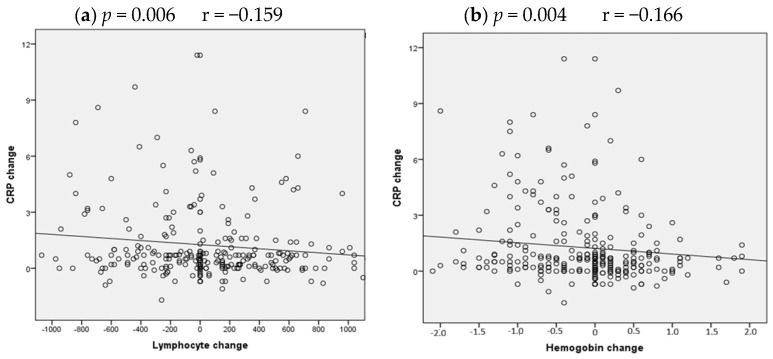
(**a**) Spearman’s correlation analysis was applied to estimate relationships between CRP changes and hematological parameters in RA patients. Scatter plot correlations of CRP with lymphocyte count change (**a**) and hemoglobin change (**b**) after 12 week treatment of methotrexate. The linear regression equation for (**a**) is: ΔCRP = 1.108 − 0.001(ΔLymphocyte), R^2^ = 0.029, *p* = 0.015. For (**b**): ΔCRP = 1.108 − 0.222(ΔHemoglobin), R^2^ = 0.015, *p* = 0.044. Correlation coefficients and *p*-values are presented in every scatter plot. *p* values < 0.05 were considered statistically significant.

Spearman’s correlation analysis examined the relationship between changes in disease activity scores and changes in hematological parameters (Table 5, Figure 1 and Figure 2). A weak inverse correlation was found between the change in DAS28-CRP and the change in lymphocyte count (r = −0.177, *p* = 0.002), as well as with the change in RDW (r = −0.147, *p* = 0.011). Regarding changes in inflammation, the reduction in CRP correlated weakly with a reduction in neutrophil count (r = 0.125, *p* = 0.031) and with a reduction in both lymphocyte count (r = −0.159, *p* = 0.006) and hemoglobin level (r = −0.166, *p* = 0.004).

To further investigate these relationships, linear regression analysis was performed with ΔDAS28-CRP as the dependent variable and changes in all hematological parameters as independent variables. The full model, including all hematological parameter changes, explained 8.7% of the variance in DAS28-CRP improvement (R^2^ = 0.087, adjusted R^2^ = 0.055, F(10, 289) = 2.756, *p* = 0.003). Among the parameters, only changes in lymphocyte count (B = −0.00024, 95% CI: −0.00038 to −0.00010, *p* = 0.001) and RDW (B = −0.004, 95% CI: −0.008 to −0.001, *p* = 0.006) emerged as significant independent predictors. Changes in NLR (*p* = 0.052) and PLR (*p* = 0.056) showed trends toward significance but did not reach the conventional threshold. The regression equation derived from this model was: ΔDAS28-CRP = 0.589 − 0.00024(ΔLymphocyte) − 0.004(ΔRDW). Detailed regression parameters are presented in Supplementary Table S4.

Similar linear regression analysis was performed with ΔCRP as the dependent variable. The model explained 8.5% of the variance in CRP improvement (R^2^ = 0.085, adjusted R^2^ = 0.053, F(10, 289) = 2.683, *p* = 0.004). Changes in lymphocyte count (B = −0.001, 95% CI: −0.0018 to −0.0002, *p* = 0.015) and hemoglobin (B = −0.222, 95% CI: −0.439 to −0.006, *p* = 0.044) emerged as significant independent predictors of CRP reduction. The regression equation was: ΔCRP = 1.108 − 0.001(ΔLymphocyte) − 0.222(ΔHemoglobin). Detailed regression parameters are presented in Supplementary Table S5. Notably, severe multicollinearity was observed for ΔNLR (VIF = 2626.38) and ΔPLR (VIF = 2619.41), which is expected as these are derived variables and does not affect the interpretation of the significant predictors.

### 3.4. Diagnostic Performance of Hematological Parameters

ROC curve analyses were conducted to evaluate the discriminatory ability of hematological parameter changes for treatment response.

**Figure 3:**
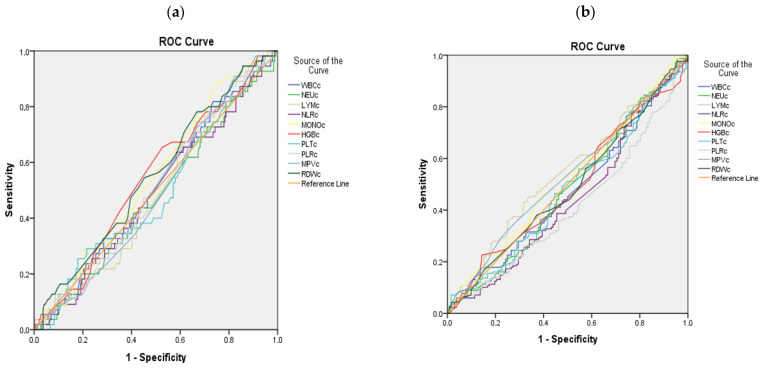
(**a**) Receiver operating characteristic (ROC) curves comparing the discriminatory ability of hematological parameter changes to distinguish remission. (**b**) ROC curves comparing the discriminatory ability of hematological parameter changes to distinguish low disease activity (LDA).

For either remission or LDA, any hematological parameter change showed significant discriminatory power (all AUC values close to 0.5, *p* > 0.05) (Table 6, Figure 3).

## 4. Discussion

This retrospective study comprehensively evaluated the longitudinal changes in hematological parameters and their relationship with treatment outcomes in DMARD-naïve RA patients initiating MTX. Our key findings are: MTX treatment induced significant alterations in multiple hematological parameters, including reductions in white blood cell, neutrophil, platelet, and hemoglobin levels, alongside an increase in RDW. While changes in lymphocyte count and RDW showed weak but statistically significant correlations with improvement in DAS28-CRP, no hematological parameter emerged as a robust independent predictor or diagnostic biomarker for remission or LDA in multivariate logistic regression or ROC analyses. Baseline clinical disease activity measures, specifically DAS28-CRP, swollen joint count, and CRP, remained the strongest predictors of 12-week treatment response. Importantly, these findings were consistent across both seropositive and seronegative patients, as demonstrated in our stratified analysis.

The observed hematological shifts are consistent with the known pharmacological and immunomodulatory effects of MTX. As a folate antagonist, MTX exerts anti-proliferative and anti-inflammatory actions through multiple pathways, including inhibition of nucleotide synthesis, modulation of adenosine release, and suppression of nuclear factor kappa-B (NF-κB) and Janus kinase/signal transducer and activator of transcription (JAK/STAT) signaling [19,20,21,22]. These mechanisms collectively reduce the production of proinflammatory cytokines such as TNF-α, IL-6, and IL-1β, which are pivotal in RA pathogenesis and also regulate hematopoiesis [23].

The reduction in WBC and neutrophil counts likely reflects the dampening of systemic inflammation and possibly a direct myelosuppressive effect of MTX, even with folate supplementation. The decrease in hemoglobin, coupled with an increase in RDW, may signify a dual influence: partial resolution of inflammation-driven anemia of chronic disease alongside MTX-induced subtle suppression of erythropoiesis or altered folate metabolism [15,24]. Elevated RDW, a marker of erythrocyte size heterogeneity, has been consistently associated with active inflammation in RA and may also be influenced by MTX’s impact on folate pathways and mean corpuscular volume (MCV) [25,26]. Interestingly, the weak but significant inverse correlation between improvement in DAS28-CRP and an increase in lymphocyte count (or a lesser decrease) suggests that favorable treatment response may mitigate inflammation-associated lymphopenia, aligning with the concept of immunomodulation restoring hematopoietic homeostasis [4].

Our findings align with prior research reporting reductions in NLR, PLR, and systemic immune-inflammation index following RA treatment [27]. However, in contrast to some cross-sectional studies suggesting that hematological indices such as NLR, PLR, and RDW could distinguish active RA from remission [28], our longitudinal, multivariate analysis did not support their utility as independent predictive biomarkers for MTX response. This discrepancy may stem from differences in study design. Previous reports were often cross-sectional, comparing patients with active disease to those in remission at a single time point, which establishes association but not prediction. Many also lacked pretreatment baselines, did not control for concurrent therapies, or were underpowered to adjust for clinical confounders. Our longitudinal design, with rigorous multivariate adjustment, provides a more robust test of their predictive value for future response, rather than just their association with current disease activity.

While our previous cross-sectional study revealed distinct hematological profiles in drug-naïve RA patients, characterized by elevated NLR, PLR, and RDW, and reduced MPV, the capacity of these parameters to reflect disease activity was limited [29]. Extending these findings, the present longitudinal evaluation before and after MTX treatment confirmed that hematological parameters offer no significant advantage over established disease activity markers in monitoring therapeutic response.

Importantly, our subgroup analyses revealed no significant differences in baseline hematological parameters, disease activity, or treatment outcomes based on gender or seropositivity status. This indicates that these factors did not confound our primary analyses and suggests that early MTX response may be similar across these subgroups in DMARD-naïve patients, an observation that merits further investigation given inconsistent literature on sex and serostatus as predictors of MTX efficacy [30,31,32].

The most critical implication of our study is that while MTX induces measurable hematological changes that loosely correlate with inflammatory attenuation, these parameters lack the specificity and strength required to serve as standalone prognostic tools in clinical practice. ROC analyses demonstrated that neither baseline values nor 12-week changes in any hematological index achieved satisfactory diagnostic accuracy for predicting remission or LDA. Although baseline PLR and RDW reached statistical significance, their AUCs were below 0.6, indicating poor discriminative capacity.

Thus, our data reinforce the continued primacy of composite clinical measures, such as DAS28-CRP, joint counts, and CRP, for monitoring early treatment response. Hematological parameters appear to be epiphenomena of systemic inflammation and its modulation rather than specific mediators of clinical response. This may reflect the pathogenic heterogeneity of RA, where hematological responses are insufficiently uniform across patients to function as reliable biomarkers.

It is also important to consider the broader context of the disease continuum. Our study focuses on patients at the point of DMARD initiation, which may represent a relatively late stage in the biological evolution of RA. The “pre-RA” phase, characterized by systemic autoimmunity and subclinical inflammation in the absence of clinical arthritis, is an area of intense investigation. It is plausible that hematological parameters, as markers of systemic inflammation, might show different trajectories or have greater predictive value during this earlier phase. This is particularly relevant for seronegative patients, who often face diagnostic delays due to the limitations of current classification systems and may have a longer period of undifferentiated inflammatory symptoms before meeting RA criteria. Future prospective studies should investigate hematological changes in individuals at risk for RA to determine if these simple biomarkers can aid in predicting the transition to clinically apparent disease.

Several limitations warrant consideration. The retrospective, single-center design introduces potential selection bias and unmeasured confounding. Our cohort consisted exclusively of DMARD-naïve patients, which may limit generalizability to those with prior DMARD exposure or more established disease. The 12-week follow-up period is relatively short for assessing sustained remission; longer observation might reveal different associations, particularly for parameters like RDW, which may be influenced by prolonged MTX use and folate metabolism. Additionally, we lacked complete data on folate supplementation dosage and duration, which could modulate MTX’s hematological effects. Despite these limitations, the study’s strengths include its well-defined cohort, comprehensive panel of hematological parameters, longitudinal design with a true baseline, and the use of robust multivariate analysis to adjust for confounders.

## 5. Conclusions

In conclusion, MTX treatment in DMARD-naïve RA patients induces significant hematological alterations reflective of its immunosuppressive and anti-inflammatory actions. While changes in parameters such as lymphocyte count and RDW weakly correlate with disease activity improvement, they do not possess sufficient predictive strength to replace or meaningfully augment conventional clinical disease activity indices for prognostication. Future prospective studies with longer follow-up, standardized folate supplementation, and integrated multi-omics approaches may help determine whether composite models incorporating serial hematological and clinical data can enhance predictive accuracy and personalize treatment monitoring in RA.

## Figures and Tables

**Table 1:** Baseline characteristics of study population.

Variable	
Gender (*n*, %)	Female: 217 (72.6%) Male: 82 (27.4%)
Age (median, IQR)	57 (47–65)
Symptom duration (years) (median, IQR)	0.5 (0.3–1)
Seropositive RA (*n*, %)	212 (70.7%)
TJC (median, IQR)	4 (3–6)
SJC (median, IQR)	3 (2–5)
VAS (mean, SD)	6.0 ± 0.75
DAS28-CRP (median, IQR)	3.59 (3.2–4.08)
DAS28-CRP (week 12) (median, IQR)	3.04 (2.9–3.27)
Change in DAS28-CRP (mean, SD)	0.59 ± 0.33
CRP (mg/dL) (median, IQR)	1.06 (0.7–2.2)
CRP (mg/dL) (week 12) (median, IQR)	0.5 (0.3–1)
Change in CRP (mean, SD)	1.26 ± 2.08
Remission (*n*, %)	55 (18.3%)
LDA (*n*, %)	168 (56%)

RA; rheumatoid arthritis, TJC; tender joint count, SJC; swollen joint count, VAS; visual analog scale, DAS28; disease activity score 28, CRP; C-reactive protein, LDA; low disease activity, SD; standard deviation, IQR; interquartile range.

**Table 2:** Comparison of baseline and 12th week values of hematological parameters.

	Week 0	Week 12	*p*	Mean Change-SD
WBC (cells/µL), (Median, IQR)	8280 (6772.5–8280)	7800 (6430–9692.5)	0.025	297.83 ± 2116.76
NEU (cells/µL), (Median, IQR)	5230 (3752.5–6517.5)	4785 (3692.5–6295)	0.026	247.57 ± 1658.10
LYM (cells/µL), (Median, IQR)	2225 (1660–2690)	2205 (1722.5–27,309)	0.763	−5.38 ± 606.47
NLR (Median, IQR)	2.34 (1.73–3.0)	2.13 (1.67–2.89)	0.114	1.98 ± 31.87
MONO (cells/µL), (Median, IQR)	600 (480–740)	590 (460–740)	0.478	13.63 ± 207.19
HGB (g/dL) (Median, IQR)	12.9 (11.8–13.9)	13 (12–14.07)	0.001	−0.15 ± 1.13
PLT (10^3^ cells/µL), (Median, IQR)	295 (242–353)	271 (218–320)	0.000	54,327.07 ± 119,766.29
PLR (Median, IQR)	139.79 (107.42–171.88)	131.35 (101.01–171.21)	0.011	106.31 ± 1696.28
MPV (fL), (Median, IQR)	10.2 (9.6–10.8)	10.1 (9.6–10.8)	0.751	−0.64 ± 10.53
RDW (%) (Median, IQR)	13.6 (13–14.87)	14.3 (13.6–15.9)	0.000	−1.09 ± 11.77

WBC; White blood cell count, NEU: neutrophil, LYM; lymphocyte, NLR: neutrophil/lymphocyte ratio, MONO; monocyte, HGB; hemoglobin, PLT: platelet, PLR; platelet/lymphocyte ratio, MPV; mean platelet volume, RDW; red cell distribution width, IQR; interquartile range, SD; standard deviation. *p* values < 0.05 were considered statistically significant.

**Table 3:** Comparison of hematological parameters, disease activity, and treatment outcomes by gender and seropositivity status.

Variable	Gender	Seropositivity
	*p* Value	*p* Value
WBC	0.700	0.884
NEU	0.862	0.887
LYM	0.389	0.137
NLR	0.853	0.114
MONO	0.335	0.580
HGB	0.422	0.812
PLT	0.602	0.600
PLR	0.611	0.074
MPV	0.099	0.894
RDW	0.330	0.257
DAS28-CRP (baseline)	0.063	0.793
CRP (baseline)	0.099	0.182
Change in DAS28-CRP (week 12)	0.350	0.744
Chande in CRP (week 12)	0.539	0.918
Remission	0.195	0.217
LDA	0.367	0.111
WBC change	0.700	0.884
NEU change	0.862	0.887
LYM change	0.389	0.137
NLR change	0.853	0.114
MONO change	0.335	0.580
HGB change	0.422	0.812
PLT change	0.602	0.600
PLR change	0.611	0.074
MPV change	0.099	0.894
RDW change	0.330	0.257

WBC; White blood cell count, NEU: neutrophil, LYM; lymphocyte, NLR: neutrophil/lymphocyte ratio, MONO; monocyte, HGB; hemoglobin, PLT: platelet, PLR; platelet/lymphocyte ratio, MPV; mean platelet volume, RDW; red cell distribution width, DAS28; disease activity score 28, CRP; C-reactive protein, LDA; low disease activity. *p* values < 0.05 were considered statistically significant.

**Table 4:** Univariate and multivariable logistic regression analyses of factors associated with remission and LDA in RA patients who received 12 weeks of methotrexate treatment.

	Univariate Analysis				Multivariate Analysis					
Variable	Remission		LDA		Remission			LDA		
	*p*	95% CI	*p*	95% CI	*p*	OR	95% CI	*p*	OR	95% CI
Age	0.086	−0.005–0.000	0.102	−0.001–0.008						
Gender	0.853	−0.096–0.079	0.624	−0.100–0.166						
Symptom duration	0.192	−0.003–0.015	0.360	−0.020–0.007						
Seropositivity	0.553	−0.108–0.058	0.296	−0.059–0.193						
TJC	0.010	−0.101–0.014	0.053	−0.001–0.132	0.571	0.819	0.411–1.632			
SJC	0.010	−0.103–0.014	0.005	0.030–0.165	0.316	10.639	0.624–4.307	0.000	1.803	1.34–2.42
VAS	0.696	−0.053–0.079	0.463	−0.138–0.063						
DAS28-CRP	0.000	0.686–1.152	0.002	−0.916–−0.208	0.000	9826.703	179.78–537,099.4	0.003	0.168	0.052–0.538
CRP	0.000	−0.121–−0.059	0.000	0.050–0.145	0.005	0.452	0.261–0.782	0.000	1.47	1.21–1.79
WBC change	0.157	0.000–0.000	0.290	0.000–0.000						
NEU change	0.160	0.000–0.000	0.827	0.000–0.000						
LYM change	0.457	0.000–0.000	0.309	0.000–0.000						
NLR change	0.748	−0.060–0.083	0.306	−0.139–0.044						
MONO change	0.408	0.000–0.000	0.168	−0.001–0.000						
HGB change	0.512	−0.056–0.028	0.432	−0.032–0.075						
PLT change	0.654	0.000–0.000	0.734	0.000–0.000						
PLR change	0.757	−0.002–0.001	0.317	−0.001–0.003						
MPV change	0.674	−0.0050.003	0.431	−0.008–0.003						
RDW change	0.729	−0.004.003	0.173	−0.001–0.008						

WBC; White blood cell count, NEU: neutrophil, LYM; lymphocyte, NLR: neutrophil/lymphocyte ratio, MONO; monocyte, HGB; hemoglobin, PLT: platelet, PLR; platelet/lymphocyte ratio, MPV; mean platelet volume, RDW; red cell distribution width, LDA; low disease activity, OR; odds ratio, CI; confidence interval. *p* values < 0.05 were considered statistically significant.

**Table 5:** Correlation analysis of disease activity and hematological parameters changes after 12 week treatment.

		WBC Change	NEU Change	LYM Change	NLR Change	MONO Change	HGB Change	PLT Change	MPV Change	PLR Change	RDW Change
DAS28 change	*p*	0.809	0.453	0.002	0.467	0.273	0.100	0.750	0.478	0.725	0.011
	r	0.014	0.044	−0.177	−0.042	−0.063	−0.095	0.019	−0.041	0.020	−0.147
CRP change	*p*	0.281	0.031	0.006	0.723	0.893	0.004	0.150	0.718	0.633	0.197
	r	0.062	0.125	−0.159	−0.021	0.008	−0.166	0.083	−0.021	0.028	−0.075

DAS28; disease activity score 28, CRP; C-reactive protein, WBC; White blood cell count, NEU: neutrophil, LYM; lymphocyte, NLR: neutrophil/lymphocyte ratio, MONO; monocyte, HGB; hemoglobin, PLT: platelet, PLR; platelet/lymphocyte ratio, MPV; mean platelet volume, RDW; red cell distribution width. *p* values < 0.05 were considered statistically significant.

**Table 6:** ROC curve analysis of hematological indices as key factors in differentiating remission and LDA after 12 week methotrexate treatment in RA patients.

	Remission			LDA		
Variable	AUC	*p*	95% CI	AUC	*p*	95% CI
WBC	0.509	0.834	0.427–0.591	0.469	0.350	0.403–0.534
NEU	0.477	0.593	0.394–0.560	0.477	0.492	0.411–0.543
LYM	0.496	0.931	0.416–0.576	0.540	0.231	0.475–0.606
NLR	0.492	0.851	0.409–0.574	0.440	0.072	0.374–0.505
MONO	0.549	0.260	0.470–0.627	0.522	0.504	0.457–0.588
HGB	0.542	0.325	0.461–0.624	0.488	0.720	0.422–0.554
PLT	0.490	0.815	0.405–0.574	0.473	0.418	0.407–0.539
PLR	0.512	0.787	0.431–0.593	0.411	0.008	0.346–0.475
MPV	0.492	0.847	0.414–0.570	0.525	0.450	0.460–0.591
RDW	0.551	0.236	0.469–0.633	0.487	0.693	0.421–0.553

WBC; White blood cell count, NEU: neutrophil, LYM; lymphocyte, NLR: neutrophil/lymphocyte ratio, MONO; monocyte, HGB; hemoglobin, PLT: platelet, PLR; platelet/lymphocyte ratio, MPV; mean platelet volume, RDW; red cell distribution width, AUC; area under curve, CI; confidence interval. *p* values < 0.05 were considered statistically significant.

## Data Availability

The data that support the findings of this study are available from the corresponding author [E.D.IMRAK], upon reasonable request.

## Supplementary Materials

The following supporting information can be downloaded at: https://www.mdpi.com/article/10.3390/biomedicines14030625/s1, Supplementary Table S1: Baseline demographic, clinic and laboratory parameters of seropositive and seronegative RA patients; Supplementary Table S2: Hematological parameters changes at week 12 in seropositive and seronegative RA patients; Supplementary Table S3: Collinearity diagnostics for multivariate logistic regression models; Supplementary Table S4: Linear regression analysis of hematological parameter changes as predictors of DAS28-CRP improvement; Supplementary Table S5: Linear regression analysis of hematological parameter changes as predictors of CRP improvement.
