# Supplementary material for: Retrospective Analysis of Hematological Parameter Changes in DMARD-Naive Rheumatoid Arthritis Patients Treated with Methotrexate: Correlation with Disease Activity and Treatment Outcomes
Source: Biomedicines. 2026 Mar 11;14(3):625. doi: 10.3390/biomedicines14030625 (PMC13024487; doi:10.3390/biomedicines14030625)
Supplement: Supplementary file 1 [file biomedicines-14-00625-s001.zip › biomedicines-4188229-supplementary.pdf]

Supplemantary Table 1: Baseline demographic, clinic and laboratory parameters of seropositive and seronegative RA patients

Variable	Seropositive RA (n=217)	Seronegative RA (n=82)	p
Age (years) (median,IQR)	57 (47-64)	56 (48-65)	0.931
Gender (n,%)	153 (70%) female<br>64 (30%)male	59 (71%) female<br>23 (29%) male	0.463
Symptom duration (years) (median,IQR)	0.5 (0.3-1)	0.5 (0.2-1)	0.003
TJC (median,IQR)	4 (3-6)	4 (2-6)	0.067
SJC (median,IQR)	3 (2-5)	3 (2-5)	0.896
VAS (median,IQR)	6 (5-6.75)	6 (6-6)	0.574
CRP (mg/dL) (median,IQR)	1.87±0.7<br>1 (0.7-1.9)	2.24±2.45<br>1.3 (0.7-2.8)	0.182
DAS28-CRP	3.59 (3.24-4.07)	3.58 (3.16-4.09)	0.793
Remission (n,%)	36 (16.5%)	19 (23.1%)	0.217
LDA (n,%) (median,IQR)	124 (57.1%)	44 (53.6%)	0.111
WBC (cells/ µL), (Median, IQR)	8320 (6792.5-9827.5)	8190 (6715-10812.5)	0.364
NEU (cells/ µL), (Median, IQR)	5250 (3175-6445)	5095 (3767.5-6717.5)	0.523
LYM (cells/ µL), (Median, IQR)	2175 (1615-2617.5)	2260 (1852.5-2750)	0.179
NLR (Median, IQR)	2.37 (1.77-2.95)	2.08 (1.69-3.32)	0.602
MONO (cells/ µL), (Median, IQR)	590 (470-720)	610 (492.5-797.5)	0.179
HGB (g/dl) (Median, IQR)	13 (11.9-13.97)	12.65 (11.7-13.67)	0.219
PLT (10^3cells /µL), (Median, IQR)	288.5 (242.25-347.75)	300 (239.75-359.75)	0.415
PLR (Median, IQR)	141.17 (108.7-171.88)	135.64 (102.91-172.13)	0.517
MPV (fL), (Median, IQR)	10.3 (9.7-10.8)	10 (9.4-10.7)	0.074
RDW (%) (Median, IQR)	13.6 (12.9-14.7)	13.6 (13.1-15)	0.375

RA; rheumatoid arthritis, TJC; tender joint count, SJC;swollen joint count, VAS; visual analogue scale, DAS28; disease activity score 28, CRP; C reactive protein, LDA; low disease activity, WBC; White blood cell count, NEU: neutrophil, LYM; lymphocyte, NLR: neutrophil/ lymphocyte ratio, MONO; monocyte, HGB; hemoglobin, PLT: platelet, PLR; platelet/lymphocyte ratio, MPV; mean platelet volume, RDW; red cell distribution width, IQR; interquartile range. p values<0.05 were considered statistically significant.

Supplemantary Table 2: Hematological parameters changes at week 12 in seropositive and seronegative RA patients

Variable	Seropositive RA (n=217)	Seronegative RA (n=82)	p
WBC change (cells/ µL), (Median, IQR)	0 (-795-1332.5)	0 (-817.5-1280)	0.884
NEU change (cells/ µL), (Median, IQR)	0 (-647.5-1067.5)	0 (-695-902.5)	0.887
LYM change (cells/ µL), (Median, IQR)	0 (-230-330)	0 (-297.5-210)	0.137
NLR change (Median, IQR)	0 (-0.37-0.49)	0.08 (-0.21-0.61)	0.114
MONO change (cells/ µL), (Median, IQR)	0 (-100-107.5)	0 (-97.5-90)	0.580
HGB change (g/dl) (Median, IQR)	0 (-0.7-0.3)	0 (-0.57-0.2)	0.812
PLT change (10^3cells /µL), (Median, IQR)	13500 (-16750-70750)	19500 (-12750-82250)	0.600
PLR change (Median, IQR)	0 (-21.07-23.67)	7.2 (-4.19-37.7)	0.072
MPV change (fL), (Median, IQR)	0 (-0.3-0.3)	0 (-0.3-0.27)	0.894
RDW change (%) (Median, IQR)	-0.7 (-1.37-0)	-0.8 (-1.47-0)	0.257

CRP change (mg/dL) (median,IQR)	0.6 (0.1-1.37)	0.7 (0-1.75)	0.918
DAS28-CRP change (Median, IQR)	0.54 (0.34-0.81)	0.53 (0.32-0.75) 1.37±2.35	0.744

RA; rheumatoid arthritis, TJC; tender joint count, SJC;swollen joint count, VAS; visual analogue scale, DAS28; disease activity score 28, CRP; C reactive protein, LDA; low disease activity, WBC; White blood cell count, NEU: neutrophil, LYM; lymphocyte, NLR: neutrophil/ lymphocyte ratio, MONO; monocyte, HGB; hemoglobin, PLT: platelet, PLR; platelet/lymphocyte ratio, MPV; mean platelet volume, RDW; red cell distribution width, IQR; interquartile range. p values<0.05 were considered statistically significant.

Supplemantary Table 3: Collinearity diagnostics for multivariate logistic regression models

Variable	Tolerance	VIF
Age	0.891	1.122
Symptom duration	0.934	1.071
Seropositivity	0.912	1.096
TJC	0.672	1.489
SJC	0.654	1.529
CRP	0.693	1.443
DAS28-CRP	0.581	1.721

VIF; Variance Inflation Factor, TJC; tender joint count, SJC;swollen joint count, CRP; C reactive protein DAS28; disease activity score 28.

Supplemantary Table 4: Linear regression analysis of hematological parameter changes as predictors of DAS28-CRP improvement

Variable	Unstandardized Coefficients	Std error	Standardized Coefficient	t	p	95%CI
ΔWBC	2.015E-05	0.000	0.073	1.149	0.252	0.000-0.000
ΔNEU	1.454E-05	0.000	0.044	0.588	0.557	0.000-0.000
ΔLYM	-0.00024	0.00007	-0.240	-3.423	0.001	-0.00038—0.0001
ΔNLR	-0.058	0.030	-5.609	-1.947	0.052	-0.117-0.001
ΔMONO	-8.761E-05	0.000	-0.055	-0.830	0.407	0.000-0.000
ΔHGB	-0.007	0.017	-0.025	-0.416	0.677	-0.042-0.028
ΔPLT	-3.138E-08	0.000	-0.011	-0.190	0.849	0.000-0.000
ΔPLR	0.001	0.001	5.511	1.916	0.056	0.000-0.002
ΔMPV	0.001	0.002	0.04	0.701	0.484	-0.000-3-0.005
ΔRDW	-0.004	0.002	-0.159	-2.788	0.006	-0.008—0.001

DAS28; disease activity score 28, WBC; White blood cell count, NEU: neutrophil, LYM; lymphocyte, NLR: neutrophil/ lymphocyte ratio, MONO; monocyte, HGB; hemoglobin, PLT: platelet, PLR; platelet/lymphocyte ratio, MPV; mean platelet volume, RDW; red cell distribution width, CI;confidence Interval. p values<0.05 were considered statistically significant.

R:0.295, R^2:0.087, adjusted R^2:0.055, standard error of the estimate: 0.32076

F(10, 289)= 2.756, p:0.003

Supplemantary Table 5: Linear regression analysis of hematological parameter changes as predictors of CRP improvement

Variable	Unstandardized Coefficients	Std error	Standardized Coefficient	t	p	95%CI
ΔWBC	2.262E-05	0.0001	0.023	0.204	0.838	0.000-0.0002
ΔNEU	0.0002	0.0001	0.200	1.603	0.110	0.000-0.0004
ΔLYM	-0.001	0.0004	-0.171	-2.436	0.015	-0.0018--0.0002
ΔNLR	-0.284	0.188	-4.356	-1.511	0.132	-0.655-0.086
ΔMONO	0.0002	0.001	0.012	0.177	0.860	-0.001-0.002
ΔHGB	-0.222	0.110	-0.121	-2.022	0.044	-0.439- -0.006
ΔPLT	8.959E-07	0.000	0.052	0.860	0.390	0.000-0.000
ΔPLR	0.005	0.004	4.298	1.492	0.137	-0.002-0.012
ΔMPV	0.011	0.011	0.054	0.937	0.349	-0.012-0.033
ΔRDW	-0.014	0.010	-0.080	-1.409	0.160	-0.034-0.006

CRP; C reactive protein, WBC; White blood cell count, NEU: neutrophil, LYM; lymphocyte, NLR: neutrophil/ lymphocyte ratio, MONO; monocyte, HGB; hemoglobin, PLT: platelet, PLR; platelet/lymphocyte ratio, MPV; mean platelet volume, RDW; red cell distribution width,CI; confidence Interval. p values<0.05 were considered statistically significant.

R:0.291, R^2:0.085, adjusted R^2:0.053, standard error of the estimate: 2.02438

$F(10, 289) = 2.683$, $p: 0.004$
